# Integrated Reproductive and Cytogenetic Assessment of Infertility in a Captive Jaguar (*Panthera onca*)

**DOI:** 10.1002/vms3.71192

**Published:** 2026-10-18

**Authors:** Marie Altmanová, Julie Strand, Kathrine Thejll Kirchhoff, Zuzana Majtánová

**Affiliations:** ^1^ Laboratory of Fish Genetics, Institute of Animal Physiology and Genetics Czech Academy of Sciences Liběchov Czech Republic; ^2^ Department of Ecology Faculty of Science Charles University Prague Czech Republic; ^3^ Department of Animal and Veterinary Sciences ‐ ANIVET Gut and host health Aarhus University Aarhus Denmark; ^4^ Randers Regnskov Randers Denmark; ^5^ Evidensia Dania Hinnerup Dyrehospital Hinnerup Denmark

**Keywords:** ART, conservation, ex situ conservation, Felidae, fluorescence in situ hybridization, semen quality

## Abstract

**Background:**

Ex situ conservation of threatened felid species requires a thorough understanding of reproductive biology and realistic evaluation of assisted reproductive techniques (ART). In jaguars (*Panthera onca*), however, knowledge of reproductive limitations in captivity remains scarce.

**Objectives:**

On the basis of a comprehensive single‐timepoint assessment, this study applied an integrated clinical, endocrinological, semen analysis and cytogenetic approach to investigate persistent infertility in a captive jaguar breeding pair recommended for reproduction within the European Association of Zoos and Aquaria (EAZA) programme.

**Methods:**

Both animals underwent comprehensive reproductive and health assessments, including genital examination, ultrasonography, haematological and hormonal profiling. Semen quality was evaluated following electroejaculation, and cytogenetic screening was performed to detect potential chromosomal abnormalities using conventional staining, banding techniques and fluorescence in situ hybridization.

**Results:**

Cytogenetic analyses revealed a standard felid karyotype (2*n* = 38) in both individuals, with no evidence of aneuploidy or structural chromosomal rearrangements. Clinical and endocrinological assessments of the female did not identify abnormalities likely to impair fertility. In contrast, semen evaluation of the male revealed markedly reduced ejaculate volume, low sperm concentration, low sperm motility and a high proportion of morphologically abnormal spermatozoa, consistent with severe teratozoospermia.

**Conclusions:**

Taken together, these findings indicate that although genetic and general clinical contraindications for reproduction were absent, semen quality represents a critical limiting factor for successful natural breeding. Under the observed reproductive profile, ART feasibility requires functional validation through sperm selection and competence assays. This study highlights the value of combining cytogenetic and reproductive assessments to support evidence‐based decision‐making in captive breeding programmes and provides novel cytogenetic data for the jaguar.

## Graphical abstract

Integrated reproductive and cytogenetic assessment of infertility in a captive jaguar pair identified normal chromosomal status in both individuals, whereas impaired semen quality in the male likely represents a major limiting factor for reproductive success under the observed conditions.

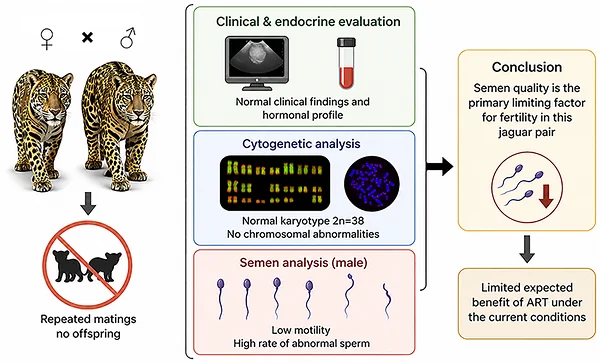

## 1 Introduction

Understanding the factors that influence reproductive biology is of critical importance for the management of endangered species in captivity. Jaguars (*Panthera onca*) are, like other large carnivores, threatened by human‐modified landscapes; due to habitat loss and fragmentation, depletion of prey and direct human interference, they have disappeared from approximately 54% of their original range (Paviolo et al. 2016; Sanderson et al. 2002). Jaguars are listed as Near Threatened by the IUCN, yet the species has already been recorded as locally extinct or critically endangered in certain areas, including the Atlantic Forest, one of the Earth's recognized biodiversity hotspots (Paviolo et al. 2016; Sanderson et al. 2002). Gathering comprehensive knowledge about the species is therefore an urgent priority in terms of future conservation strategies and captive breeding programmes.

In captivity, breeding programmes for jaguars are coordinated by zoo associations worldwide with the aim of retaining the highest possible genetic diversity. The European Association of Zoos and Aquariums (EAZA) coordinates population management for species held in member institutions through the European Endangered Species Programme (EEP). Each EEP is overseen by a coordinator assisted by a Species Committee, who are collectively responsible for conducting demographic and genetic analyses and preparing future management plans (EAZA Conservation Programmes 2019). On the basis of the most recent available data from 2025 EEP report (Lassen 2025), the European population consisted of 42 males and 47 females in 41 different zoos that were participating in the programme. The average generation time was 7 years. During 2025, two cubs were born of the 16 pairs recommended for breeding in 2025. The goal of the Jaguar EEP is to maintain a self‐sustaining population; population growth is not an explicit objective. Due to incomplete pedigree data for many individuals, mean kinship and inbreeding coefficients cannot be applied universally for pairing decisions. Addressing the paucity of pedigree data through genetic profiling of all EEP individuals, for example, using microsatellite or SNP‐based approaches, has been identified as a priority for improving kinship‐based pairing decisions (Rueda‐Zozaya et al. 2016).

Despite coordinated management, the reproductive biology of jaguars remains comparatively understudied relative to other felids, such as lions and cheetahs, and published data remain limited (Beisiegel et al. 2012; Durant et al. 2004; Packer et al. 2005; Paviolo et al. 2016; Sanderson et al. 2002). A recent comprehensive review of both in situ and ex situ jaguar reproduction underscored that fundamental knowledge gaps persist and continue to hamper the success of captive management and assisted reproductive technologies, particularly regarding male fertility, ovarian physiology and the practical application of ART in conservation programmes (Deco‐Souza et al. 2024).

### 1.1 Reproductive Characteristics of Jaguars

Jaguars are solitary animals with a territorial social structure. Home range size varies markedly across habitats and prey availability, ranging from tens to several hundred square kilometres in different populations (Gonzalez‐Borrajo et al. 2017; Moreira‐Ramírez et al. 2021; Sanderson et al. 2002). In their natural habitat, males and females occupy separate ranges, interacting only during mating. In captivity, jaguars are housed in considerably smaller enclosures that attempt to simulate natural conditions within the constraints of available space. Lighting, temperature and feeding regimes vary among institutions, and the degree to which the natural habitat is replicated differs accordingly. Three specific factors are recognized as influencing reproductive success: genetics, nutrition and environmental conditions (Lasley et al. 1994; Morato et al. 2001). Non‐domestic felids are known to reproduce poorly in captivity, either due to chronic stress or as a consequence of small breeding populations and the associated loss of genetic variation (Brown et al. 1994; Comizzoli et al. 2000). Small and isolated populations are particularly susceptible to inbreeding depression and increasing homozygosity resulting from mating between closely related individuals (Brown et al. 1994; Comizzoli et al. 2000). A variety of additional mediating factors that reduce fertility have been proposed (Morato et al. 2004; Thongphakdee et al. 2020); for example, physiological stress leads to an increase in serum cortisol concentration, which is known to reduce testosterone levels in male jaguars (Morato et al. 2004) and in other wild felids such as the cheetah (Lindburg et al. 1993).

Regarding nutritional requirements, diets consisting exclusively of red beef may be deficient in several essential minerals and vitamins. Low concentrations of vitamins A and E in particular can compromise spermatogenesis and lead to testicular degeneration (Morato et al. 1999; Rodrigues da Paz et al. 2006). Dietary supplementation with vitamins (A, D, E, B and folic acid) and minerals (calcium, phosphorus, iron, copper, iodine and manganese) to jaguars with a diet consisting of only red meat resulted in a 35% decrease in primary sperm abnormalities and an overall improvement in semen quality over a supplementation period of 6–12 months (Morato et al. 1999; Rodrigues da Paz et al. 2006). These findings highlight the importance of nutritional management as a modifiable factor influencing male reproductive performance in captive jaguars.

The female jaguar has a seasonally influenced reflex ovulation mechanism, comparable to that of the domestic cats (Rodrigues da Paz 2012). Ovulation is triggered by a mating‐induced neuronal stimulus and a reflex release of gonadotrophin‐releasing hormone (GnRH) from the hypothalamus, followed by a subsequent surge of luteinizing hormone (LH) from the anterior pituitary gland (Brown 2006). Jaguars are also known to ovulate spontaneously throughout the year (Barnes et al. 2015; Jimenez Gonzalez et al. 2017). A more recent study confirmed that ovulation can be induced by non‐copulatory stimuli in jaguars in the absence of copulation (Jorge‐Neto et al. 2020). The reproductive parameters for captive female jaguars are summarized in Table 1. Characterization of oestrous cycle length and puberty onset in captive females has been described in detail, with mean inter‐oestrous intervals and the onset of puberty varying between individuals and facilities (Viau et al. 2020).

**TABLE 1:** Reproductive characteristics for captive female jaguars (*Panthera onca*) (Rodrigues da Paz 2012).

Oestrus (days)	Oestrus cycle (days)	Gestation (days)	Number of cubs
12.0 ± 1	47.2 ± 5.4	90–111	1–2

The non‐pregnant luteal phase in felids is characterized by elevated faecal progestin concentrations persisting for more than 20 days following ovulation in the absence of fertilization, with a mean duration of 29.6 ± 2.6 days (Barnes et al. 2015). Unlike in dogs and ferrets, in which luteal phase duration is similar regardless of pregnancy status, felids, including jaguars, exhibit a shorter non‐pregnant luteal phase than during pregnancy. Ovarian activity in domestic cats is related to photoperiod, whereas in wild felids it is also influenced by seasonal variation in food intake (Rodrigues da Paz et al. 2006). A study of captive female jaguars demonstrated seasonal variation in gonadal activity, with the lowest activity recorded between October and December, including periods of anoestrus (Jimenez Gonzalez et al. 2017).

No significant seasonal differences in semen quality and affection of the endocrine‐testicular function have been reported in male jaguars (Jimenez Gonzalez et al. 2017; Morato et al. 1999, 2004); however, a difference has been observed between dry and wet seasons, with a significantly higher faecal androgen concentrations during the wet period, a finding hypothesized to reflect greater food availability and its positive effect on reproductive function (Morato et al. 2004). Earlier studies suggested poorer semen quality in captive jaguars relative to free‐ranging individuals (Morato et al. 2001). However, Ribeiro de Araujo et al. (2018) reported that total sperm output differed significantly between groups, whereas motility and sperm morphology did not. Together, these findings indicate that the effects of captivity on semen quality may vary among populations and management conditions. Morphological abnormalities were dominated by primary sperm defects, with head and tail defects being the most frequently observed (Silva et al. 2019). Recent advances in jaguar andrology have further characterized sperm ultrastructure, mitochondrial activity and cryopreservation parameters, providing a more detailed biological basis for interpreting semen quality data in this species (Jorge‐Neto et al. 2023; Santos et al. 2022; Silva et al. 2019).

Recent advances in jaguar‐specific ART have substantially extended the practical toolkit available for conservation breeding. Laparoscopic ovum pick‐up (LOPU) combined with exogenous gonadotropin stimulation has proven safe and effective for recovering large numbers of viable oocytes from jaguars (Jorge‐Neto et al. 2023). Heterologous in vitro fertilization (IVF) using frozen‐thawed jaguar semen and domestic cat oocytes has demonstrated that jaguar spermatozoa can retain fertilizing capacity even when morphologically compromised, with embryo development to the blastocyst stage achieved using appropriately selected cryopreservation extenders (Santos et al. 2022). Most significantly, the first live jaguar cub produced via laparoscopic artificial insemination (AI) was recently reported, validating species‐adjusted hormonal synchronization protocols and optimized AI timing for population management (Vansandt et al. 2026). Despite these advances, the application of ART in European captive wild felid populations remains largely reactive, employed primarily in cases of diagnosed infertility rather than as a routine management tool, and is constrained by logistical challenges, high costs and the need for specialized expertise (Prochowska et al. 2022).

### 1.2 Cytogenetic Characteristics of Felidae

The first karyotypes of 22 felids were described in 1968 (Wurster‐Hill and Benirschke 1968), and a few years later eight additional species were added, bringing the total number up to 30 species which had been studied with the usage of conventional staining (Wurster 1969; Wurster‐Hill and Benirschke 1968). Knowledge of chromosome evolution within the order Carnivora was subsequently advanced by the pioneering G‐banding studies of Wurster‐Hill and collaborators (Wurster‐Hill and Bush 1980; Wurster‐Hill and Centerwall 1982; Wurster‐Hill and Grey 1975). The family Felidae is well known for its highly conserved karyotypes with reported diploid chromosome numbers of 2*n* = 38 and 2*n* = 36, where 2*n* = 38 is predominant (Eroglu 2017; Hsu et al. 1963; O'Brien et al. 2006; Suedmeyer et al. 2003; Tanomtong et al. 2008; Wurster‐Hill and Centerwall 1982; Wurster‐Hill and Grey 1973). The standard karyotype is represented by six chromosome groups, based on their size and centromeric position (Eroglu 2017; O'Brien et al. 2006; Wurster‐Hill and Grey 1973) and previous studies have confirmed the jaguar karyotype to be 2*n* = 38 (Eroglu 2017; Ledesma et al. 2004). Despite these early discoveries, cytogenetic research within Felidae has remained limited, and for most species only basic karyotype descriptions are available (Tanomtong et al. 2008). Genome‐wide homology maps have been described only for African lion (*Panthera leo*), clouded leopard (*Neofelis nebulosa*) and domestic cat (*Felis catus*) by using dog chromosome specific probes (Tian et al. 2004; Yang et al. 2000), further confirming the high degree of karyotype conservation within the family. Advanced molecular cytogenetic analyses, including C‐banding, CMA_3_/DAPI staining and fluorescence in situ hybridization (FISH), remain absent from the published literature for jaguars specifically, representing a significant gap in the characterization of this species’ genome. The integration of cytogenetic screening into captive breeding programmes has demonstrated clear utility in other felids; chromosomal disorders and disorders of sexual development have been revealed by karyotyping in Siberian tigers and Eurasian lynx, directly informing breeding management decisions. The application of such techniques to jaguars is therefore both timely and warranted, particularly given the reproductive challenges documented in captive populations, and in the broader context of ART implementation across European zoos (Prochowska et al. 2022).

### 1.3 Objectives

Despite coordinated management within the EAZA EEP, a significant proportion of captive jaguar breeding pairs fail to produce offspring, and the underlying causes of infertility in many cases remain unidentified. Although recent advances in ART have demonstrated technical feasibility for several key reproductive interventions (Jorge‐Neto et al. 2023; Santos et al. 2022; Vansandt et al. 2026), successful application of these technologies depends on accurate diagnosis of individual reproductive limitations and realistic evaluation of breeding potential. Furthermore, although cytogenetic abnormalities are recognized contributors to infertility in other felid species, the absence of comprehensive cytogenetic characterization in jaguars represents a critical knowledge gap that limits diagnostic capacity in conservation breeding programmes (Deco‐Souza et al. 2024).

The objective of this study was to investigate the factors potentially contributing to persistent infertility in a captive jaguar breeding pair selected for reproduction within the EAZA programme at Randers Regnskov, Denmark. Despite regular mating behaviour, no pregnancies had been recorded in this pair for a period of 4 years. To determine the underlying causes of infertility and evaluate the prognosis and prospects for future reproduction, we applied an integrated multi‐approach framework comprising external and internal clinical examination, including ultrasonography; semen collection and analysis; endocrinological and haematological profiling; and comprehensive cytogenetic screening using conventional staining, banding techniques and FISH. Specifically, this study aimed to (i) identify potential clinical or endocrinological factors limiting fertility in both individuals through comprehensive reproductive and health assessments; (ii) evaluate male reproductive capacity through detailed analysis of ejaculate characteristics, sperm motility and sperm morphology; (iii) detect potential chromosomal abnormalities in both individuals and provide the first molecular cytogenetic characterization of *P. onca*; (iv) integrate findings across diagnostic modalities to identify the primary factors limiting reproductive success and to provide evidence‐based recommendations for breeding management and potential ART application. This study provides important multidisciplinary insight into jaguar physiology and cytogenetics, substantially deepening our understanding of this species’ reproductive biology and offering a comparative reference for future ART applications in European captive populations.

## 2 Materials and Methods

### 2.1 Animals and Ethical Approval

A male and a female jaguar intended for breeding were included in this study. Both individuals were born in captivity in 2014 and have been at Randers Regnskov, a tropical zoological facility in Denmark, since the age of 5 months. The animals were selected for breeding by the EAZA coordinator on the basis of a low inbreeding coefficient. They were housed together and had access to both outdoor and indoor covering a total of 900 m^2^. Fresh water was available ad libitum, and both animals were fed twice weekly with a variety of red meat. The female (body weight 61 kg) received 5 kg and the male (body weight 83 kg) received 7 kg of meat per feeding, supplemented with a commercial vitamin and mineral preparation formulated for carnivores (Kasper Faunafood, JH Woerden, Holland), added at the manufacturer's recommended rate of 1% of the diet. The female exhibited a non‐seasonal oestrus cycle on 54–62 days duration. Behavioural oestrus lasted 9–12 days, during which the pair mated multiple times per hour. The mating behaviour observed was consistent with normal species‐typical patterns. The female had never produced a litter and, to the authors’ knowledge, had never been pregnant.

All procedures were conducted in accordance with applicable national and institutional regulations governing the use of animals in research and veterinary practice.

### 2.2 Capture and Anaesthesia

The jaguars were fasted 24 h prior to anaesthesia, restrained in the indoor enclosure and chemically immobilized. The female was anesthetized by intramuscular injection of a combination of medetomidine (0.03 mg/kg, Domitor, Orion Pharma, UK), midazolam (0.2 mg/kg, B. Braun, USA), butorphanol (0.1 mg/kg, Torbugesic, Orion Pharma, UK) and ketamine (5 mg/kg, Ketaminol, MSD, Denmark). The male was anesthetized intramuscularly by intramuscular injection of medetomidine (0.09 mg/kg, Orion Pharma, UK), midazolam (0.075 mg/kg, B. Braun, USA) and ketamine (5 mg/kg, Ketaminol, MSD, Denmark). Anaesthesia was reversed in both animals by intramuscular administration of Atipemazol (0.045–0.14 mg/kg, Antisedan, Orion Pharma, UK) in muscle (Ribeiro de Araujo et al. 2018; Zambelli et al. 2007).

### 2.3 Reproductive Assessment of the Female

#### 2.3.1 Clinical Examination and Ultrasonography

The female was examined 14 days after her last oestrous period. External genitals were inspected visually and the vagina was explored by digitally palpation. Transabdominal ultrasonographic examination of the uterus and ovaries was performed using a portable ultrasound unit (Esaote MyLab Delta, Kruuse, Langeskov, Denmark) (Figure 1). A routine health assessment, including dental examination, was also performed, whereas the animal was under anaesthesia.

**FIGURE 1:**
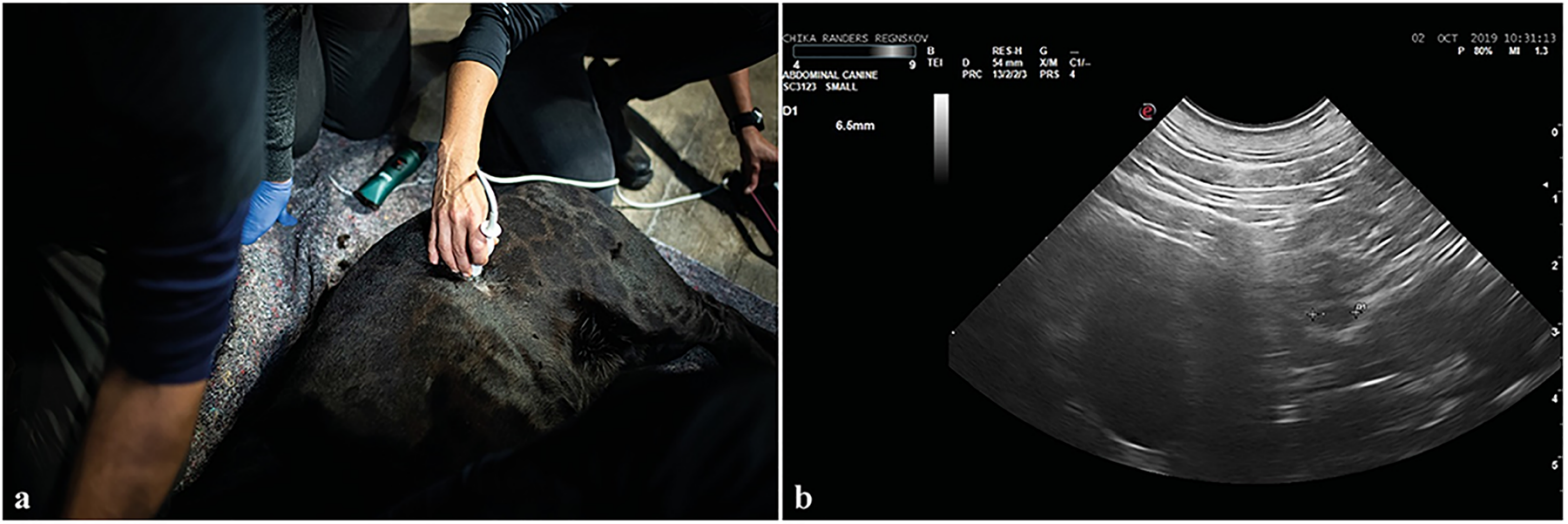
Clinical examination and ultrasound of female jaguar (Panthera onca). (a) Transabdominal ultrasound of the uterus and ovaries and (b) ultrasound of ovaries, including measurements of ovarian follicle.

#### 2.3.2 Endocrinology and Haematological Screening

Blood samples were collected by venipuncture from the jugular vein into plastic collection tubes (Kruuse, Odense, Denmark). Serum was obtained by centrifugation at 5000 RPM for 5 min. Progesterone (P4) concentrations were measured by fluorescence enzyme immunoassay according to the manufacturer's instructions using the AIA‐360 analyser (TOSOH, Tessenderlo, Belgium), with an inter‐assay coefficient of variation 2.8%. This analyser has been shown to produce results comparable with chemiluminescence‐based methods and has been validated for P4 measurements in dogs, cats and horses (Diquelou et al. 2015). It should be noted, however, that the AIA‐360 has not been formally validated for use in jaguars or other neotropical felids; results should, therefore, be interpreted with appropriate caution, and comparison with species‐validated assays is recommended in future studies (Deco‐Souza et al. 2024). The remaining serum was submitted for analysis of a standard biochemistry panel, haematological parameters and thyroid profile at a commercial diagnostic laboratory (IDEXX, Berlin, Germany). Where jaguar‐specific reference intervals were available, these were used for result interpretation; for parameters lacking published jaguar reference values, reference intervals from the domestic cat were applied (Homola et al. 2025), as detailed in Table 2.

**TABLE 2:** Blood serum parameters from both male and female jaguars.

Parameter	Female	Male	Reference interval/n
Glucose	8.73 mmol/L	8.01 mmol/L	7.77 ± 3.0 mmol/L/n = 203
Creatinine	165 µmol/L	216 µmol/L	168 ± 58 µmol/L/n = 202
Carbamid	6.9 mmol/L	7.4 mmol/L	8.9 ± 3.6 mmol/L/n = 206
Carbamid/Creatinin	10	8	No reference interval
Phosphate	1.54 mmol/L	1.52 mmol/L	1.58 ± 0.39 mmol/L/n = 167
Calcium	2.24 mmol/L	2.31 mmol/L	2.45 ± 0.23 mmol/L/n = 197
Total protein (TP)	72 g/L	73 g/L	74 ± 7 g/L/n = 178
Albumin (ALB)	33 g/L	31 g/L	34 ± 4 g/L/n = 149
Globolin (GLOB)	39 g/L	42 g/L	40 ± 8 g/L/n = 148
ALB/GLOB	0.8	0.7	No reference interval
Alanine aminotransferase (ALT)	24 U/L	40 U/L	55 ± 26 U/L/n = 161
Alkaline phosphatase (ALKP)	16 U/L	11 U/L	32 ± 33 U/L/n = 194
Gamma‐glutamyltransferase (GGT)	1 U/L	0 U/L	2 ± 2 U/L/n = 82
Total bilirubin	3 µmol/L	4 µmol/L	3 ± 2 µmol/L/n = 176
Cholesterol	3.62 mmol/L	3.84 mmol/L	6.29 ± 1.6 mmol/L/n = 178
Amylase	2242 U/L	1966 U/L	301.2 ± 149.1 U/L/n = 63
Lipase	112 U/L	113 U/L	4.44 ± 3.3 U/L/n = 25
Thyroxine, free (fT4)	15.6 pmol/L	5.9 pmol/L	9.0–33.5 pmol/n = 1*
Thyroxine, total (tT4)	23.2 nmol/L	11.6 nmol/L	27 ± 19 nmol/L/n = 18
Total testosterone	Not analysed	4.74 ng/mL	0.3 ± 4*

*Note*: Reference values represent mean ± standard deviation for captive jaguar (*Panthera onca*) (AZA Jaguar Species Survival Plan 2026). Parameters lacking jaguar‐specific reference values are compared against domestic cat reference intervals to Homola et al. (2025) (indicated with *).

### 2.4 Reproductive Assessment of the Male

#### 2.4.1 Clinical Examination and Ultrasonography

The penis, prepuce and testes were examined by visual inspection and palpation. Testicular dimensions (length and two breadths) were recorded using a calliper, and testicular volume was calculated using the equation for an ellipsoid: *V* = 4/3 × *π* × *L*/2 × *B*1/2 × *B*2/2, where *L*, *B*1 and *B*2 represent the length and two breadths of the testis (Harcourt et al. 1995). Ultrasonographic examination of the testes was performed using the same portable unit described in Section 2.3.1 (Esaote MyLab Delta, Kruuse, Langeskov, Denmark). A routine health assessment, including dental examination, was also performed, whereas the animal was under anaesthesia (Figure 2).

**FIGURE 2:**
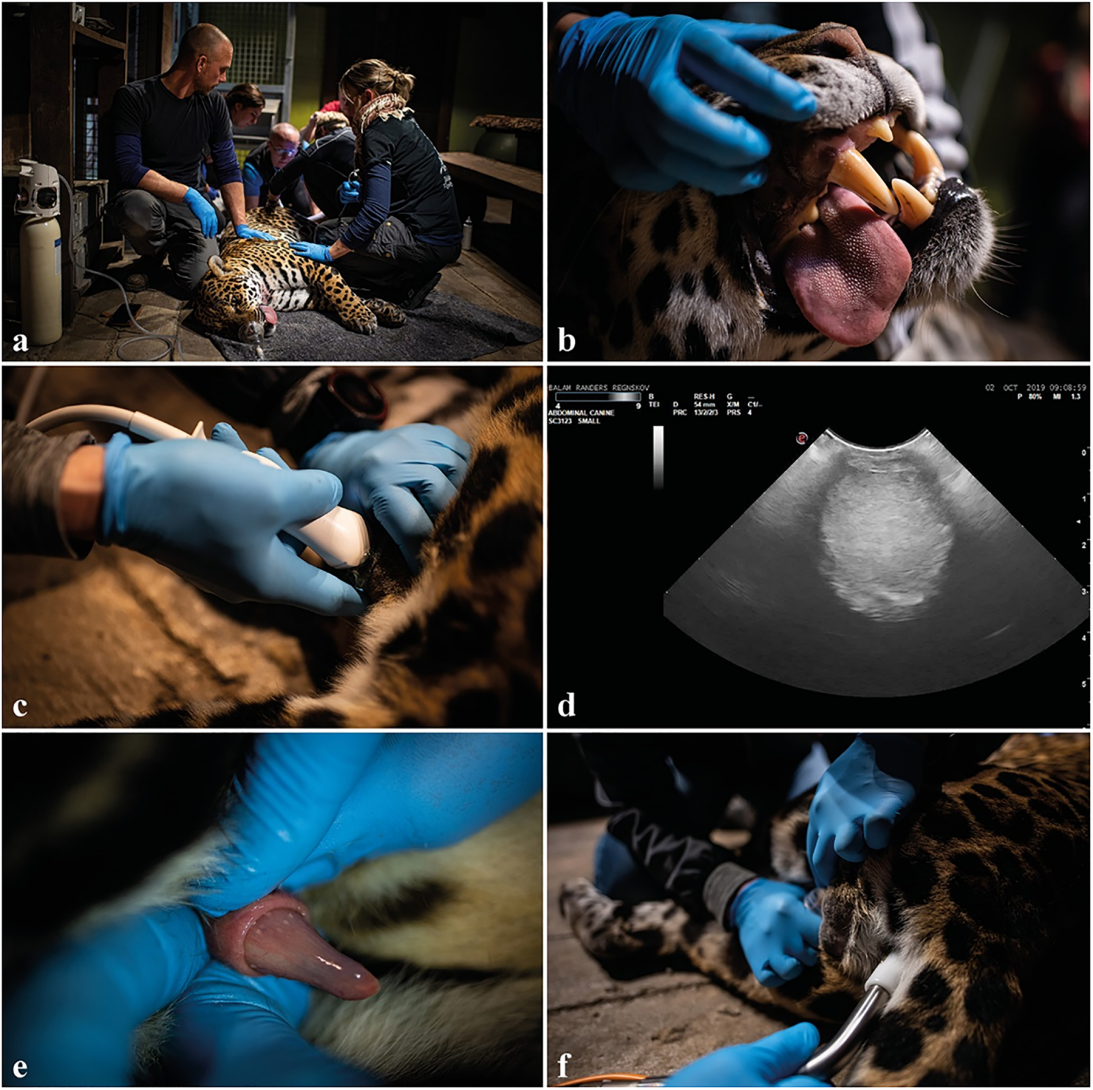
Overview of general clinical examination and ultrasound of male jaguar (Panthera onca). (a and b) General health check. (c and d) Measuring, examination and ultrasound of testicles. (e) Examination of penis and prepuce. (f) Semen collection by probe and an electroejaculator.

#### 2.4.2 Semen Collection and Analysis

Semen was collected by electroejaculation under general anaesthesia, as described in Section 2.2. Electroejaculation was selected over urethral catheterization given the animal's temperament and to ensure collection under standardized conditions consistent with previously published protocols for jaguars (Deco‐Souza et al. 2024; Morato et al. 1999; Rodrigues da Paz 2012; Zambelli et al. 2007). A rectal probe (diameter: 2.6 cm; length: 14.5 cm) connected to an electroejaculator (Minitube GmbH, Tiefenbach, Germany) was used to deliver the electrical stimulation (Figure 2). The electrode was lubricated with mineral oil and introduced into the rectum with the copper strips positioned ventrally and in firm contact with the pelvic plexus region. A standardized sequence of 90 electrical stimuli, divided into three sets with 5‐min rest intervals between sets, was applied. The first set consisted of 30 stimuli at 2, 3 and 4 V; the second of 30 stimuli at 3, 4 and 5 V; and the third of 30 stimuli using 10 stimuli each at 5, 6 and 7 V, as described in previous studies (Morato et al. 1999; Rodrigues da Paz 2012; Zambelli et al. 2007). Ejaculate evaluation was performed immediately following collection. A total of three semen fractions were collected during the electroejaculation procedure and pooled for analysis. Semen was collected in Eppendorf tubes (Kruuse, Langeskov, Denmark) with a few drops of prewarmed isotonic saline and maintained at 35°C throughout examination. Samples were subsequently diluted with isotonic saline to a final concentration of 30 × 10^6^ spermatozoa/mL to allow standardized assessment of sperm motility. Sperm motility and the degree of forward progressive motility were assessed subjectively under phase‐contrast light microscopy at 40× magnification (Leica, Wetzlar, Germany). It is acknowledged that subjective motility assessment introduces inter‐observer variability; computer‐assisted sperm analysis (CASA) would provide a more objective and reproducible evaluation and is recommended for future studies (De Araújo et al. 2021). Nevertheless, CASA equipment was not available at our facility. Ejaculate parameters recorded included volume, pH, estimated percentage of progressively motile spermatozoa, percentage of non‐viable spermatozoa and sperm concentration. Sperm concentration (per mL) and the percentage of non‐viable cells were determined using a NucleoCounter SP‐100 (Chemometec, Allerød, Denmark). Morphological assessment was performed on Spermac‐stained semen smears (Spermac Stain, Minitube GmbH, Tiefenbach, Germany) by counting 200 spermatozoa at 100× magnification (oil immersion), and the percentage of morphologically abnormal cells was recorded.

#### 2.4.3 Endocrinology and Haematological Screening

Blood samples were collected and processed as described Section 2.3.2. Serum was submitted to IDEXX (Berlin, Germany) for analysis of a standard biochemistry panel, haematological parameters, total thyroxin (T4) and serum testosterone concentration. All measured parameters are listed in Table 2.

### 2.5 Cytogenetic Assessment of Male and Female

#### 2.5.1 Chromosomal Preparation and Staining

Mitotic chromosome spreads were prepared from peripheral blood leukocytes cultures. For both individuals, 500 µL of whole blood was cultured at 37°C for 64 h in 5 mL of DMEM medium (Sigma‐Aldrich) supplemented with 10% foetal bovine serum (Gibco, Carlsbad, CA, USA), 3% phytohaemagglutinin M solution (Gibco), 2 mM l‐glutamine (Sigma‐Aldrich), 100 µg/mL lipopolysaccharide (Sigma‐Aldrich, St. Louis, MO, USA), 100 U/mL of penicillin and 100 µg/mL of streptomycin (Gibco). Approximately 45 min before harvesting, 35 µL of colcemid solution (10 µg/mL stock solution, Roche) was added to the medium to synchronize and stop the cell division at metaphase. Cells were subjected to hypotonic treatment with 0.075 M KCl at 37°C for 30 min, followed by three rounds of fixation in Carnoy's fixative (3:1, methanol:acetic acid). Part of leukocyte cell cultures were treated by ethidium bromide (EBr) to produce elongated chromosomes suitable for subsequent G‐banding treatment. EBr was used in final concentration 50 µg per sample, added 2 h before harvesting, and cell cultures were harvested as described above.

To determine chromosome number and morphology in both individuals, microscopic slides were stained with 5% Giemsa solution for 10 min. C‐banding, visualizing blocks of constitutive heterochromatin, was performed according to the protocol of Sumner (1972) with slight modifications. Specifically, the slides were aged at 65°C for 1 h, immersed in 0.4% HCl for 20 min, treated with 5% Ba(OH)_2_ solution for 3 min at 45°C and incubated in 2× SSC for 1 h at 65°C, then rinsed in distilled water and counterstained with Vectashield DAPI anti‐fade medium (Vector Laboratories, Burlingame, CA, USA). G‐banding was performed sensu Seabright (1971). Briefly, 1‐day‐old slides were aged at 60°C for 12 h, then treated in Coplin jar including 2.5 mg of trypsin in 45 mL of Sörensen's buffer for 50 s at 26°C, rinsed by Sörensen's buffer, stained in 4.8% Giemsa solution (4 mL of Giemsa stain, 2.4 mL of methanol and 80 mL of Sörensen's buffer), rinsed by distilled water and air‐dried. To reveal GC‐rich regions of chromosomes, chromomycin A_3_ (CMA_3_) staining was performed as described by Sola et al. (1992), using Vectashield DAPI anti‐fade medium as a counterstaining and mounting reagent. Silver staining of nucleolar organizer regions (Ag‐NOR) was performed according to Howell and Black (1980).

#### 2.5.2 FISH With Telomeric Probe and rDNA Genes

The topology of the telomeric repeats (TTAGGG)*_n_* and ribosomal DNA (rDNA) genes was analysed by FISH. Telomeric FISH was performed using a Cy3‐labelled peptide nucleic acid (PNA) probe according to the manufacturer's instructions (Telomere PNA FISH Kit/Cy3, Dako, Denmark). For rDNA FISH, a probe containing partial sequences of 28S rDNA genes was produced as described by Majtánová et al. (2019) and was indirectly labelled with biotin‐16‐dUTP (Roche, Mannheim, Germany) by PCR reamplification under the same conditions as the original PCR reaction. Labelled PCR products were precipitated, dissolved in hybridization buffer and processed for hybridization and detection as described by Symonová et al. (2015). The biotin‐dUTP‐labelled probe was detected by FITC‐Streptavidin (Invitrogen, San Diego, CA, USA), and the slides were mounted with Vectashield DAPI anti‐fade medium (Vector Laboratories).

#### 2.5.3 Microscopy and Image Analyses

Chromosomal preparations were examined by a ZEISS Axio Imager.Z2 epifluorescence microscope equipped with a CoolCube 1 camera (MetaSystems, Altlussheim, Germany). Grey‐scale images were analysed using IKAROS and ISIS imaging software (MetasyStems, Altlussheim, Germany). Digital images from FISH experiments were pseudocoloured (red for Cy3‐labelled PNA telomeric probe and green for Invitrogen FITC‐Streptavidin) and merged using Adobe Photoshop software, version CS5. For CMA_3_/DAPI staining, the CMA_3_ signal was pseudocoloured in the red and the DAPI signal in the green to enhance the contrast between these two types of signals. Chromosomal spreads after C‐banding were taken in the fluorescent regime and inverted. The chromosomal categories were classified according to standardized feline karyotype nomenclature (Cho et al. 1997; O'Brien et al. 2006; Wurster‐Hill and Centerwall 1982; Wurster‐Hill and Grey 1973).

## 3 Results

### 3.1 Reproductive Assessment of Female

External genitalia were normally formed for the species and appropriately positioned to permit mounting and intromission by the male. On digital exploration, a circumferential mucosal fold was identified at the vestibulo‐vaginal junction; this structure admitted only a single finger and may represent a normal anatomical variant or a mild congenital stenosis. Although this finding was not considered likely to impair vaginal stimulation sufficient for reflex ovulation induction, its potential to contribute to dystocia in a future pregnancy warrants prospective monitoring and further characterization by vaginoscopy under anaesthesia. Ultrasonographic examination revealed no pathological uterine changes. Small follicular structures (2–3 mm) were present on both ovaries. Serum progesterone exceeded 40 ng/mL (upper assay limit), confirming non‐pregnant luteal phase consistent with recent ovulation and active luteal function. Haematological, biochemical and thyroid parameters are provided in Table 2 (see Section 2.3.2). Shared biochemical findings for both individuals are reported in Section 3.3.

### 3.2 Reproductive Assessment of Male

By palpation, the testicles were of normal texture, with a length 4.3 cm of the left and 4.1 cm of the right testicle. Ultrasound showed no pathological changes on both testicles. Penis was easy to protrude, and the length was 2.5 cm with penile spines. Results of basic chemistry, haematology, total thyroxin (T4) and serum testosterone are provided in Table 2. The male (body weight: 83 kg) showed low testicular volume, low sperm concentration, markedly reduced sperm motility and a high proportion of morphological defects. Semen parameters and spermatozoa morphological defects are identified and are listed in total (%) in Tables 3 and 4. Serum testosterone concentration was 4.74 ng/mL (Table 2). As no species‐specific reference interval has been established for jaguars, interpretation was based on the available domestic cat reference range (Homola et al. 2025). Semen parameters are summarized in Tables 3 and 4. Shared biochemical findings for both individuals are reported in Section 3.3.

**TABLE 3:** Comparison of reproductive and semen parameters of the male jaguar (*Panthera onca*) from Randers Regnskov with corresponding values reported for captive and free‐living male jaguars by Ortiz et al. (2022).

Characteristics	Randers Regnskov male	Captive jaguars	Free‐living jaguars
Bodyweight (kg)	83	53.0 ± 2.6 to 72.0 ± 11.0	95.8 ± 35.4 to 96.0 ± 7.7
Testicular volume (cm^3^)	33.7	41–6 ± 0.6 to 44.4 ± 2.0	52.4 ± 3.4
Semen volume (mL)	1.3	0.292 ± 0.326 to 8.3 ± 0.7	0.43 ± 0.262 to 4.1 ± 0.7
Sperm concentration (×10^6^/mL)	33	5.7 ± 1.1 to 2091.4 ± 1816.19	35.0 ± 21.3 to 3315 ± 1141.67
Total spermatozoa/ejaculate (×10^6^)	42.9	59.3 ± 12.8 to 316.6 ± 398.98	152.0 ± 88.0 to 1280.75 ± 578.53
Sperm motility (%)	10	57.0 ± 4.5 to 93 ± 1.5	73.0 ± 6.6–81 ± 7.4
Rate of progressive status (0–5)	2	2.8 ± 0.1 to 4.8 ± 0.1	3.5 ± 0.2 to 3.9 ± 0.54
Serum testosterone concentration (ng/mL)	4.5	3.1 ± 0.7	2.1 ± 0.8

**TABLE 4:** Spermatozoa morphological defects (%) in ejaculate for the male jaguar (*Panthera onca*) examined in this study.

Normal spermatozoa	Tail defects	Midpiece	Proximal droplets	Distal droplets	Macrocephaly	Microcephaly	Acrosome defects	Loose heads
5	45	5	0	0	9	12	15	9

### 3.3 Clinical and Haematological Findings

Both animals showed markedly elevated amylase (2242 and 1966 U/L vs. reference 301.2 ± 149.1 U/L) and lipase (112–113 U/L vs. reference 4.44 ± 3.3 U/L), alongside lower alanine aminotransferase (ALT), alkaline phosphatase (ALKP), gamma‐glutamyltransferase (GGT) and cholesterol relative to the captive jaguar reference group. Although elevated amylase and lipase are typically associated with pancreatitis, neither animal showed clinical signs of this condition. A plausible hypothesis is that both animals consumed a high‐fat meal shortly before blood collection; this should be evaluated in future assessments by implementing a standardized pre‐sampling fast of at least 12 h.

### 3.4 Cytogenetic Assessment of Male and Female

The karyotypes of studied individuals consist of 2*n* = 38 chromosomes in standard feline arrangement A1–A3, B1–B4, C1–C2, D1–D4, E1–E3, F2–F3 autosomal pairs and one pair of sex chromosomes (Figure 3a,b). No chromosomal abnormalities were detected in either individual. C‐banding revealed strongly heterochromatic pair of sex chromosomes in both sexes—two middle‐sized metacentric chromosomes in a female and one middle‐sized metacentric and one small‐sized submetacentric in a male (Figure 3c,d). Moreover, two heterochromatic blocks have been determined in pericentromeric regions of chromosome pair E1. G‐banding produced the banding pattern congruent in both male and female (Figure 3e,f), with only difference being Y chromosome with rather homogenous composition (Figure 3e). Reversed fluorescence staining (CMA_3_/DAPI) revealed banding patterns across whole chromosomes with highly GC‐rich signals in its telomeric regions (Figure 3g,h). Additional conspicuous GC‐rich signal was detected on p‐arm of chromosome pair E1. Both sexes resemble the banding pattern. Telomeric FISH with probe to test for signs of any putative chromosomal rearrangements. Telomeric FISH confirmed normal terminal distribution of (TTAGGG)*_n_* repeats with no interstitial telomeric sequences, excluding cryptic rearrangements (Figure 3i,j). FISH with 28S rDNA and AgNOR staining congruently identified p‐arm of E1 chromosome pair as an active nucleolar organizer region (Figure 3k,l; and boxes, respectively). To the authors’ knowledge, this study represents the first application of C‐banding, CMA_3_/DAPI staining and FISH with telomeric and rDNA probes in *P. onca*. These data constitute a novel molecular cytogenetic characterization of the jaguar and provide a reference for karyotype evolution within Felidae.

**FIGURE 3:**
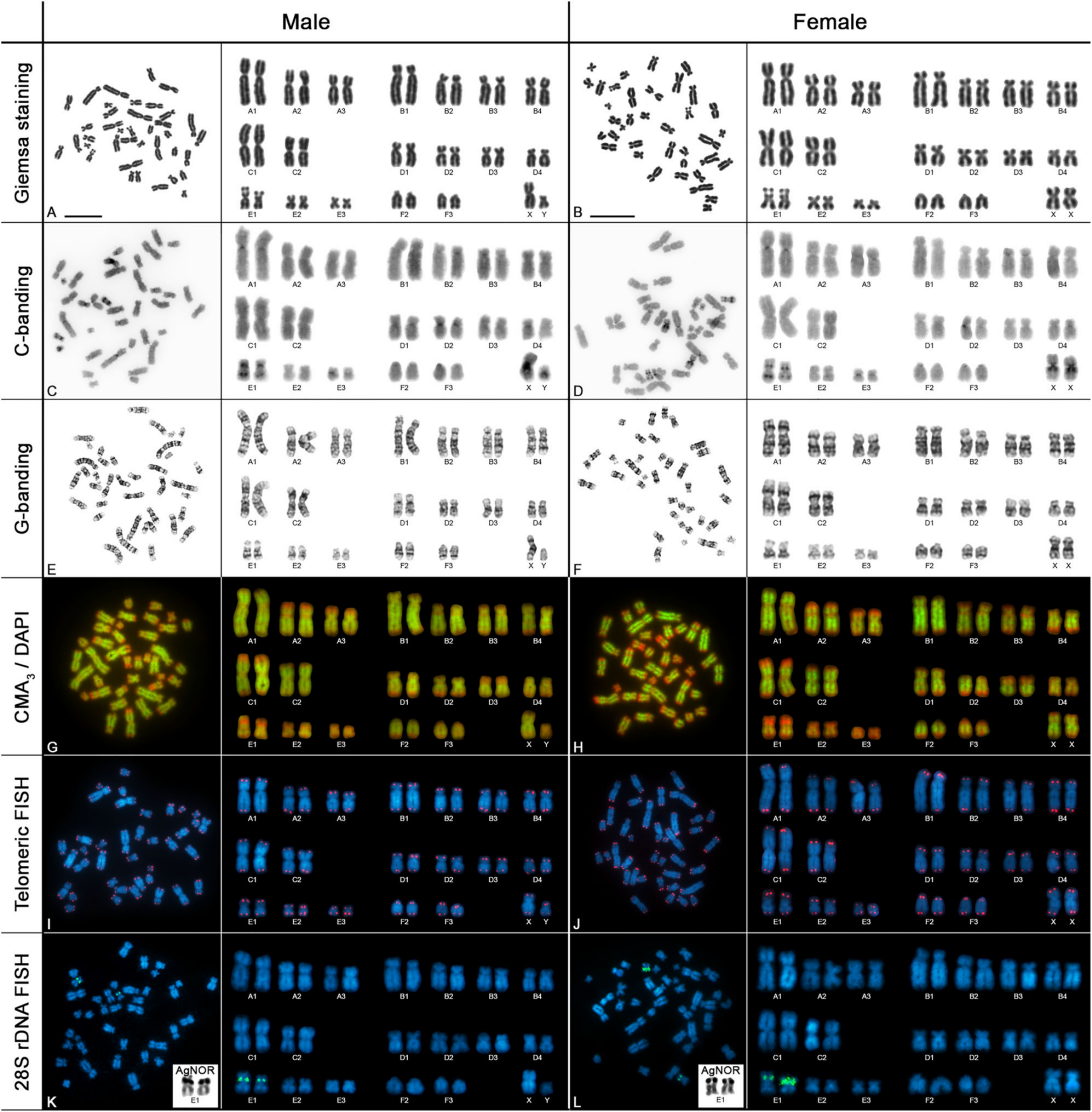
Cytogenetic screening of male and female jaguar (*Panthera onca*). Representative karyogram arranged from Giemsa‐stained chromosomes (a and b). Inverted DAPI‐stained C‐banding pattern highlighting clusters with constitutive heterochromatin located in the pericentromeric regions of chromosomal pair E1 and heterochromatinized sex chromosomes (c and d). G‐banding showing chromosome‐specific pattern (e and f). DAPI‐stained metaphase chromosomes (*green*) with signals of GC‐rich sites (*red*) (g and h). DAPI‐stained chromosomes (*blue*) with telomere repeat hybridization signals (*red*) (i and j). DAPI‐ stained metaphase chromosomes (*blue*) with 28S rDNA hybridization signals (*green*) and AgNOR signals (*black*) revealed on chromosome pair E1 (*in white box*) (k and l). Bar equals 10 µm.

## 4 Discussion

This study presents the most comprehensive reproductive and cytogenetic assessment of a captive jaguar pair reported to date, integrating clinical examination, ultrasonography, endocrinological profiling, semen analysis and molecular cytogenetic characterization. The findings identify a combination of severely reduced sperm concentration, low progressive motility and extreme teratozoospermia as the primary reproductive limitation in this male, while also providing the first advanced molecular cytogenetic characterization of *P. onca*, a contribution of foundational significance for the species. The following discussion interprets the clinical and cytogenetic findings in the context of current knowledge, addresses the implications for assisted reproductive technology (ART) and identifies key limitations and priorities for future investigation.

### 4.1 Reproductive Assessment

#### 4.1.1 Female

Clinical and endocrinological assessment of the female did not reveal any abnormalities considered likely to impair fertility under natural or assisted breeding conditions. The female's inter‐oestrous interval of 54–62 days is consistent with published values for captive jaguars (Viau et al. 2020; Wildt et al. 1979), and her hormonal profile was within the expected range for a reproductively active female. Progesterone concentrations exceeding 40 ng/mL, recorded on one occasion, are consistent with a non‐pregnant luteal phase—a physiological state documented in felids following ovulation without fertilization (Wildt et al. 1979). The detection of a non‐pregnant luteal phase in this female is noteworthy, as it confirms that ovulatory activity is occurring and that the hypothalamic–pituitary–gonadal axis is functioning, but that fertilization, and therefore implantation, is not being achieved. Given that jaguars are induced ovulators capable of ovulation triggered by non‐copulatory stimuli (Jorge‐Neto et al. 2020), the occurrence of a non‐pregnant luteal phase in the absence of successful mating outcomes further supports the interpretation that infertility in this pair is primarily attributable to male‐factor semen quality rather than female reproductive dysfunction.

A circumferential mucosal fold at the vestibulo‐vaginal junction was identified during gynaecological examination. Although this finding was not considered a primary cause of infertility under natural breeding conditions, its potential clinical significance warrants prospective monitoring. Specifically, this anatomical variant could represent a risk factor for dystocia in the event of a future pregnancy, and its significance for transcervical insemination procedures, should ART be pursued, should be evaluated by an experienced reproductive specialist prior to any such attempt. The remaining gynaecological and ultrasonographic findings were unremarkable, and the female was considered a suitable candidate for continued breeding attempts.

#### 4.1.2 Male

Clinical examination of the male revealed that he was in a good physical condition. By palpation, the testicles were of normal size and texture, and ultrasound showed no pathological changes. Penis was easily protruded and with penile spines as expected for a healthy animal. Semen was collected successfully under medetomidine‐ketamine anaesthesia as described by Silva et al. (2019). Compared to previous reports (Rodrigues da Paz 2012; Silva et al. 2019), the evaluated semen showed a small volume, a low motility and a high amount of morphological sperm defects predominantly affecting the sperm tails and heads as described by Silva et al. (2019). This pattern is consistent with the consolidated reference dataset of Deco‐Souza et al. (2024), whose Table 1 aggregates all published jaguar seminal parameters: electroejaculation in captive jaguars yields volumes of 2.0–11.0 mL, total motility of 57%–77% and 27%–81% morphologically normal spermatozoa. The present ejaculate fell below the lower bounds of all electroejaculation‐derived reference ranges for both motility and morphology reported therein, confirming the clinical severity of the semen impairment. Teratozoospermia (less than 60% of morphologically normal sperm cells) is a recognized feature of poor‐quality felid semen and has been documented across multiple captive wild felid species (Morato et al. 1999; Prochowska et al. 2022). The male examined in this study displayed only 5% morphologically normal spermatozoa with a motility of 10% (Vigor 2), representing severely compromised semen quality expected to reduce fertility to a minimal level (Jimenez Gonzalez et al. 2017; Ribeiro de Araujo et al. 2018). It is important to note, however, that teratozoospermia alone does not necessarily preclude natural fertility in jaguars: Jorge‐Neto et al. (2023) documented males with as few as 10% morphologically normal spermatozoa that successfully produced offspring. In those cases, adequate sperm concentration appeared to compensate for poor morphology. In the present male, the concurrent reduction in sperm concentration and progressive motility alongside extreme teratozoospermia represents a more compelling multifactorial basis for reproductive failure than teratozoospermia alone. Morato et al. (2001) showed that semen quality for captive jaguars is inferior compared to free‐living jaguars, findings replicated here. Serum testosterone concentration was 4.74 ng/mL. As no species‐specific reference interval has been established for jaguars, interpretation was based on the available domestic cat reference range. Although the measured value was within the reported reference range and was consistent with the presence of penile spines and normal libido, this finding should be interpreted with caution because testosterone concentrations are known to fluctuate markedly over time. Consequently, a single measurement may not accurately reflect endocrine status or fertility potential. Repeated sampling over a standardized time period would provide a more reliable assessment of testosterone secretion patterns; however, repeated immobilization was considered impractical in the present case because of the associated anaesthetic risks. Free T4 was below the domestic cat reference lower limit; however, the male showed no clinical signs of hypothyroidism, and the absence of jaguar‐specific free T4 reference intervals precludes a definitive interpretation. Reassessment using a species‐validated assay and a larger jaguar reference cohort is recommended in future evaluations.

As previously reported, the stress is a key factor negatively affecting the fertility when wild felids are kept in captivity (Lasley et al. 1994; Morato et al. 1999, 2004). None of the two animals displayed any direct stress behaviour such as restlessness, weight loss or aggressiveness. Nevertheless, considering that wild jaguars are solitary and very territorial in their natural habitat, a certain amount of stress is expected and may have affected the fertility in both animals. Morato et al. (2004) demonstrated that faecal glucocorticoid metabolite measurement is a sensitive non‐invasive tool for stress assessment in captive jaguars. The absence of faecal cortisol measurement in this study represents a significant limitation; its inclusion is strongly recommended in future assessments.

Semen evaluation revealed severe teratozoospermia, with 95% morphologically abnormal spermatozoa and only 10% progressive motility. These values are markedly below published reference thresholds for jaguars and other felids (Table 3). These values are consistent with a clinically significant impairment of natural fertilizing capacity. Although teratozoospermia alone has been shown insufficient to preclude fertility in some jaguars (Jorge‐Neto et al. 2023), the concurrent low sperm concentration and reduced progressive motility observed here represent a multifactorial reproductive compromise that collectively explains the pair's failure to conceive. The most prevalent morphological defects were midpiece abnormalities and bent or coiled tails, which are associated with mitochondrial dysfunction and impaired flagellar motility—defects that directly compromise sperm penetration capacity (Pukazhenthi et al. 2006).

The cause of severe teratozoospermia in this male remains undetermined. A single‐timepoint semen collection, as performed in this study, is insufficient to establish whether the observed semen quality reflects a chronic reproductive disorder or a transient perturbation. Best practice in andrological assessment of wild felids recommends a minimum of three to five collections, spaced at least 2 weeks apart, to account for temporal variation in semen quality (Prochowska et al. 2022). The absence of repeated collections represents a significant limitation of the present study and should be addressed in any follow‐up assessment. A limitation of the present study is that sperm morphology was recorded using predefined defect categories, without detailed quantification of individual abnormality subtypes or severity grades. Future studies should incorporate more comprehensive morphological assessments to better evaluate the potential contribution of specific sperm defects to reduced fertility in jaguars. Further investigation should also focus on chronic stress via faecal glucocorticoid analysis (de Souza et al. 2026), nutritional deficiencies or phytoestrogen exposure (Pukazhenthi et al. 2006) and molecular genetic profiling to verify genomic diversity beyond incomplete studbook records (Bauer et al. 2018). The potential influence of body condition and metabolic status on reproductive performance warrants further investigation. Future evaluations must also adopt objective CASA methodology and functional sperm assays (acrosome integrity, DNA fragmentation) to accurately define the male's fertilizing potential (Deco‐Souza et al. 2024; Silva et al. 2019). Deco‐Souza et al. (2024) have established and validated a species‐specific CASA configuration for jaguar spermatozoa using Animal Breeders II software (Hamilton Thorne), requiring a minimum of ∼20 × 10^6^ spermatozoa/mL for reliable kinematic assessment; this standardized protocol should be adopted in any future andrological re‐evaluation.

### 4.2 Assisted Reproductive Technologies

Several species of wild felids are suffering from poor semen quality due to the loss of genetic diversity and various environmental stress factors (Tanomtong et al. 2008; Thongphakdee et al. 2020). To overcome this issue, assisted reproductive techniques (ARTs) such as semen collection, sperm cryopreservation, AI, IVF, embryo cryopreservation and embryo transfer (ET) have been developed and constitute a valuable tool for conserving genetic diversity within felids (Kochan et al. 2019; Swanson and Brown 2004; Thongphakdee et al. 2020). Species‐specific knowledge for jaguars remains insufficient to optimize outcomes. As reviewed by Deco‐Souza et al. (2024), ART in jaguars is principally constrained by poor sperm freezability (post‐thaw motility consistently falls below 30% across all published protocols) and by the recognition that non‐copulatory ovulation stimuli must be factored into AI and LOPU protocols, as female ovarian response to exogenous hormones is significantly enhanced by proximity to males (Deco‐Souza et al. 2024). To be able to benefit from these techniques, basic knowledge of endocrinology and reproductive biology is crucial for successful results. Endocrine analysis from faeces has been performed in many of the South American felids, including the jaguar (Morato et al. 2004, 2001; Swanson et al. 2003; Wildt et al. 1979). For jaguars specifically, basic knowledge is still lacking and is needed to improve the success rate of ARTs. Until now, only the successful sperm cryopreservation has been performed, and results have shown the frozen spermatozoa to be functional in vitro (Rodrigues da Paz et al. 2002; Swanson et al. 2003); however, post‐thaw motility remains low across cryoprotectant protocols (Jorge‐Neto et al. 2023). Deco‐Souza et al. (2024) compiled six independent cryopreservation trials showing post‐thaw motility ranging from 4.3% ± 2.5% to 46.0% ± 7.7%, with the majority of protocols yielding values below 30% even when fresh motility exceeded 90%. Santos et al. (2022) achieved embryo production via heterologous IVF using frozen jaguar sperm and domestic cat oocytes, confirming residual fertilizing capacity despite low efficiency. For AI, the wild felids are treated with exogenous gonadotrophins, such as equine chorionic gonadotropin (eCG) and human chorionic gonadotropin (hCG), to induce the oestrus and ovulation, prior to laparoscopic intrauterine insemination. Rodrigues da Paz et al. (2004) showed that using eCG/hCG in an interval of 4 months and up to six total treatments, the ovarian responsiveness did not decrease, even though the females produced anti‐gonadotropin immunoglobulins in both ocelots and tigrinas. These results suggest that the reproductive cycle of felids can be effectively managed using this treatment regime and thereby used in ARTs without decreasing the fertility. Laparoscopic oviductal AI in jaguars using an eCG/hCG stimulation protocol has been described previously; however, reproductive success has remained limited, with no live offspring reported to date (Jimenez et al. 1999). Previous research in Brazil has been focused on using IVF in ocelots, jaguars and tigrinas. The stimulation of the ovaries by follicle stimulating hormone (FSH)/LH resulted in 25 collected follicles per female with more than 80% recovered oocytes in high quality from jaguars. Embryos were produced after IVF, but the fertilization rate was rather low with less than 25% success (Morato et al. 2000). The use of ET has not yet been performed in jaguars, but in other species like the ocelot the healthy offspring was produced by using laparoscopic transfer protocols conducted for the domestic cat (Swanson 2001, 2002).

In the present case, severely compromised semen quality substantially limits natural breeding prospects. However, it would be premature to conclude that ART offers no benefit. Sperm selection techniques (density gradient centrifugation, swim‐up) may enrich the morphologically normal, motile subpopulation and ICSI, which bypasses many barriers associated with poor sperm quality, could theoretically be explored, though it has not yet been reported in jaguars. The overall prognosis remains guarded; a definitive assessment of ART feasibility should await the results of sperm selection trials and functional assays (e.g., heterologous oocyte penetration tests), rather than being precluded on morphological assessment alone.

### 4.3 Cytogenetic Assessment

The family Felidae is well known for its highly conserved karyotypes (O'Brien et al. 2006), where the diploid number of chromosomes is 2*n* = 38, with the exception in four species of genus *Leopardus* displaying 2*n* = 36, likely evolved via chromosomal fusion (Eroglu 2017; O'Brien et al. 2006). Chromosome number and morphology represent a valuable tool in evolutionary studies and may serve also as a taxonomical trait (Eroglu 2015, 2017). For example, Eroglu (2017) determined the evolutionary relationships in 23 Felidae species by the karyotype symmetry/asymmetry index. The banding patterns within Felidae were studied by Wurster‐Hill and Centerwall (1982) and their study of 30 species distinguished 16 different karyotypic groups in felids, based on a variability in banding pattern of 10 chromosome pairs. The rest of chromosomal pair appeared to be homologous among studied feline species (Wurster‐Hill and Centerwall 1982). In contrast, O'Brien et al. (2006) reported the only obvious difference in two acrocentric chromosome pairs among the Felidae species. Despite the fact, that various chromosomal patterns found among felid species after G‐banding comparisons were later confirmed by molecular methods (Wurster‐Hill and Centerwall 1982; Wurster‐Hill and Grey 1973), the whole genome analysis and chromosome mapping will be needed to confirm the chromosome evolution within the Felidae family.

The usefulness of cytogenetics in captive breeding programmes was illustrated, for example, in Siberian tiger (*Panthera tigris altaica*) and Eurasian lynx (*Lynx lynx*) (Mastromonaco et al. 2012; Suedmeyer et al. 2003). In Siberian tiger, the cause of infertility was inspected on the basis of measuring of serum testosterone levels and by cytogenetics after biopsy. The results of testosterone measurements did not show striking difference between sterile and healthy males (1287.1 pg/mL in sterile individual, compared to values ranging from 1329.1 to 1760.1 pg/mL in three healthy males). However, after cytogenetic analyses, G‐banding revealed a chromosomal disorder, also known as Klinefelter's syndrome, displaying one extra X chromosome (39, XXY) in sterile male (Suedmeyer et al. 2003). Klinefelter's syndrome is considered as a cause of sterility in more than two‐thirds of sterile domestic felines (Suedmeyer et al. 2003). Based on karyotyping analyses in *Eurasian lynx*, Mastromonaco et al. (2012) identified a cryptorchid male that had previously been considered female. These cases demonstrate the irreplaceability of cytogenetics for animals under breeding programmes.

Our cytogenetic assessment of male and female both displayed standard chromosomal features, in terms of number and morphology, similar to already published data (Morato et al. 2000; Tanomtong et al. 2008; Wurster‐Hill and Centerwall 1982; Wurster‐Hill and Grey 1973). The karyotypes of both individuals consisted of 2n = 38 chromosomes, grouped into A1–A3, B1–B4, C1–C2, D1–D4, E1–E3, F2–F3 chromosomal pairs, and one pair of sex chromosomes. Using several banding techniques (C‐banding, CMA_3_/DAPI and G‐banding) we got congruent results in both animals and with no apparent chromosomal abnormalities in sense of the aneuploidies or chromosomal rearrangements. Our application of FISH with telomeric probe did not show any interstitial signals as signs of putative chromosomal rearrangements and revealed a normal distribution of telomeric sequences at the termini of all chromosomes in both male and female. To the authors’ knowledge, C‐banding, CMA_3_/DAPI staining and FISH with telomeric and rDNA probes have not previously been reported for *P. onca*. These results represent a novel molecular cytogenetic characterization of the jaguar karyotype and contribute to our understanding of karyotype evolution within Felidae. Regarding the comparison with Ledesma et al. (2004), who reported a jaguar karyotype differing from the standard feline arrangement with respect to centromeric index: The G‐banding patterns in the present study are broadly similar to those of Ledesma et al. (2004), and the higher resolution preparations achieved here did not support a systematic difference in centromeric index. The discrepancy may reflect differences in preparation quality, chromosome selection for measurement, or genuine population‐level polymorphism between the Argentinian individuals studied by Ledesma et al. (2004) and the European captive‐bred individuals examined here. We conclude that the present data do not provide compelling evidence to classify the jaguar karyotype as categorically distinct from the standard feline arrangement; however, broader sampling across geographically diverse populations is required to definitively resolve this question.

### 4.4 Implications for the Jaguar EEP and Future Directions

The findings of this study have several practical implications for the management of jaguars within the EAZA EEP. The identification of severely impaired semen quality as the primary reproductive limitation in this pair underscores the value of comprehensive andrological assessment as a routine component of breeding programme management. Given the documented incompleteness of jaguar EEP pedigrees and the limited number of breeding recommendations issued annually (Bauer et al. 2018), the reproductive capacity of each genetically valuable individual assumes particular importance. The recommendation for molecular genetic profiling of all EEP‐registered jaguars, using microsatellite markers or SNP arrays, remains a high priority. Accurate inbreeding coefficients and genome‐wide diversity estimates are essential for optimizing breeding recommendations and identifying individuals at elevated risk of inbreeding depression, which may manifest partly through impaired semen quality. More broadly, the present study demonstrates the value of integrating cytogenetic screening into routine reproductive assessments for endangered felids. The first molecular cytogenetic characterization of *P. onca* reported here provides foundational reference data that will be of utility for future comparative genomic studies, conservation genetic analyses and diagnostic screening of karyotypic abnormalities in captive jaguars. The adoption of similar multi‐modal assessment frameworks, combining clinical, endocrinological, andrological and cytogenetic approaches, is recommended for other undercharacterized felid species managed within ex situ conservation programmes.

## 5 Conclusion

High‐quality breeding management and a thorough understanding of reproductive biology are critical for maintaining a genetically healthy and demographically sustainable captive jaguar population within the EAZA programme (Lassen 2025). This study demonstrates the value of an integrated reproductive and cytogenetic approach for investigating infertility in captive felids in a minimally invasive manner.

Cytogenetic screening revealed no chromosomal abnormalities in either individual, and this study provides the first molecular cytogenetic characterization of *P. onca*, including the first application of C‐banding, CMA3/DAPI staining and FISH in this species, constituting a novel reference baseline for Felidae. The concurrent presence of low sperm concentration, markedly reduced progressive motility and extreme teratozoospermia in the male was identified as the primary limiting factor for natural breeding. Although teratozoospermia alone does not preclude fertility in jaguars (Jorge‐Neto et al. 2023), this multifactorial combination substantially compromises natural fertilizing capacity. Although the prospects for ART are currently constrained, sperm selection trials and functional assays should be evaluated before ART is definitively precluded (Deco‐Souza et al. 2024; Jorge‐Neto et al. 2023; Prochowska et al. 2022; Santos et al. 2022). To strengthen future breeding strategies, microsatellite‐based or SNP genotyping of all EAZA jaguar EEP individuals should be prioritized to enable mean kinship‐based pairing decisions.

## Author Contributions

**Marie Altmanová**: methodology, investigation, writing – review and editing, visualization. **Julie Strand**: methodology, investigation, writing – review and editing, visualization. **Kathrine Thejll Kirchhoff**: methodology, investigation, writing – review and editing, visualization. **Zuzana Majtánová**: methodology, investigation, writing – review and editing, visualization; supervision.

## Funding

This research did not receive any specific funding from agencies in the public, commercial or not‐for‐profit sectors. Z.M. was supported by the Czech Academy of Sciences (PPLZ L200451951), M.A. was supported by Charles University Research Centre program (UNCE/24/SCI/00). Publication costs were supported by the Frimodt‐Heineke Foundation.

## Ethics Statement

The authors confirm that the ethical policies of the journal, as noted on the journal's author guidelines page, have been adhered to and the appropriate ethical review committee approval has been received. The US National Research Council's guidelines for the Care and Use of Laboratory Animals were followed.

## Conflicts of Interest

The authors declare no conflicts of interest.

## Data Availability

The data that support the findings of this study are available from the corresponding author upon reasonable request.
